# Maternal and fetal complications associated with systemic lupus erythematosus: An updated meta-analysis of the most recent studies (2017–2019)

**DOI:** 10.1097/MD.0000000000019797

**Published:** 2020-04-17

**Authors:** Wen Rong He, Hua Wei

**Affiliations:** Department of Obstetrics and Gynecology, The First Clinical Medical College of Yangtze University, Jingzhou, Hubei, China.

**Keywords:** abortion, cesarean section, fetal complications, fetal loss, intrauterine growth retardation, low birth weight, maternal complications, pre-eclampsia, small for gestation, stillbirth, systemic lupus erythematosus

## Abstract

**Background:**

Recent guidelines provide better treatment and management of pregnancy in women with systemic lupus erythematosus (SLE). In this analysis, we aimed to systematically assess the maternal and fetal complications associated with SLE using the most recent studies (2017–2019) to obtain an updated result of the present situation.

**Methods:**

http://www.clinicaltrials.gov, MEDLINE, Cochrane Central, Web of Science, EMBASE, and Google Scholar were searched for English based studies comparing maternal and fetal complications in pregnant women with versus without SLE. Maternal and fetal complications were the endpoints in this analysis. The RevMan software 5.3 (latest version) was the most suitable analytical software for this analysis. Data were represented by risk ratio (RR) with 95% confidence interval (CI).

**Results:**

A total number of eight million eight hundred and twelve thousand two hundred seventy-two (8,812,272) participants were included in this analysis, consisting of 9696 SLE-associated pregnancy. Based on an analysis of recently published studies (2017–2019), pre-eclampsia/eclampsia was significantly higher in pregnant women with SLE (RR: 3.38, 95% CI: 3.15–3.62; *P* = .00001). SLE was also associated with an increased risk of stillbirth (RR: 16.49, 95% CI: 2.95–92.13; *P* = .001) and fetal loss (RR: 7.55, 95% CI: 4.75–11.99; *P* = .00001). Abortion (RR: 4.70, 95% CI: 3.02–7.29; *P* = .00001) and the risk for cesarean section due to complications (RR: 1.38, 95% CI: 1.11–1.70; *P* = .003) were also significantly higher in pregnant women with SLE. In addition, fetal complications including preterm birth (RR: 2.33, 95% CI: 1.78–3.05; *P* = .00001), infants who were small for gestational age (RR: 2.50, 95% CI: 1.41–4.45; *P* = .002) and infants with low birth weight (RR: 4.78, 95% CI: 3.65–6.26; *P* = .00001) were also significantly higher in newborns from mothers with SLE. Moreover, the risk of newborns who were admitted to the neonatal intensive care unit (RR: 2.79, 95% CI: 2.31–3.37; *P* = .00001), newborns with an APGAR score <7 within 1 minute (RR: 2.47, 95% CI: 1.68–3.62; *P* = .00001) and 5 minutes (RR: 3.63, 95% CI: 2.04–6.45; *P* = .0001) respectively, were significantly highly associated with SLE.

**Conclusions:**

Based on the most recent studies, we could conclude that maternal and fetal complications were significantly higher in SLE-associated pregnancy. Therefore, SLE should still be considered a severe risk factor for pregnancy.

## 1 Introduction

Autoimmune disorders affect a minor population throughout the world. However, these diseases are often associated with life-threatening complications.^[1]^ Systemic lupus erythematosus (SLE) is one among the most common autoimmune disorders affecting females of child-bearing age.^[2]^ As stated in other studies, research concerning SLE in pregnant women have often been limited to a particular ethnic group, or most of the time to a specific region.^[3,4]^ Therefore, to generalize this issue, Bundhun et al clearly demonstrated the impact of SLE on maternal and fetal outcomes through a meta-analysis including various studies from different parts of the globe and included studies which were published between the years 2001 and 2016.^[5]^ The authors clearly showed the associated adverse events with this life threatening disease throughout pregnancy and stated that special care and treatment should be provided to those women to minimize the risk of unfavorable outcomes.

Recently, new treatment strategies were incorporated in guidelines for the treatment and management of pregnant women with SLE.^[6]^ Following these updated guidelines, several new studies were published. Therefore, since the previous meta-analysis only focused on studies which were published up to the year 2016, we aimed to systematically assess the maternal and fetal complications associated with SLE using the most recent studies (2017–2019) to obtain an updated result of the actual situation.

## 2 Materials and methods

### 2.1 Search databases and search strategies

http://www.clinicaltrials.gov, MEDLINE (PubMed), Cochrane Central, Web of Science, EMBASE, and Google Scholar were searched for English based studies comparing maternal and fetal complications in pregnant women with and without SLE.

The searched terms which were used include:

(1) Systemic lupus erythematosus and pregnancy;
(2) Systemic lupus erythematosus and pregnancy outcomes;
(3) Systemic lupus erythematosus and pregnancy complications;
(4) Systemic lupus erythematosus and maternal outcomes;
(5) Systemic lupus erythematosus and fetal outcomes;
(6) Systemic lupus erythematosus and maternal complications;
(7) Systemic lupus erythematosus and fetal complications;
(8) Systemic lupus erythematosus and adverse pregnancy outcomes;
(9) Systemic lupus erythematosus and obstetrical outcomes.

The word “systemic lupus erythematosus” was also replaced by the abbreviated term “SLE.”

### 2.2 Major criteria for inclusion

Major criteria for inclusion were:

(1) Studies based on maternal and fetal outcomes in pregnant women with and without SLE;
(2) Studies which were published after the year 2016 (2017–2019);
(3) English language publications.

### 2.3 Major criteria for exclusion

Major criteria for exclusion were:

(1) Literature reviews, systematic reviews, and meta-analyses;
(2) Case studies;
(3) Relevant studies which were published in or before the year 2016;
(4) Non-English language publications;
(5) Studies that did not involve SLE and pregnancy;
(6) Studies without a control group;
(7) Studies where relevant outcomes were not reported;
(8) Duplicated studies.

### 2.4 Data extraction and quality assessment

First of all, the names and year of publication of the studies were extracted. Then the maternal and fetal complications provided in the original studies were extracted independently by the authors. Based on these endpoints, a selection was done to pick up endpoints which were most relevant and specific for this analysis. Also, the general and baseline features including the total number of SLE and non-SLE associated pregnancies, the types of study, the methodological features, and the percentage of smokers, the mean age of the females, and the number of prenatal visits were all extracted. At last, the authors also extracted the number of events associated with each complication (maternal and fetal).

During the data extraction process, any possible disagreement was discussed and resolved by consensus.

For the observational studies, the methodological quality was assessed with reference to the Newcastle–Ottawa scale^[7]^ whereby a grade A (low bias risk), B (moderate bias risk), or C (high bias risk) was allotted.

### 2.5 Outcomes reported in the selected studies

The maternal and fetal complications which were reported in the original studies have been listed in Table 1. Based on this list, a selection of outcomes was made to represent this study (as endpoints).

**Table 1:**
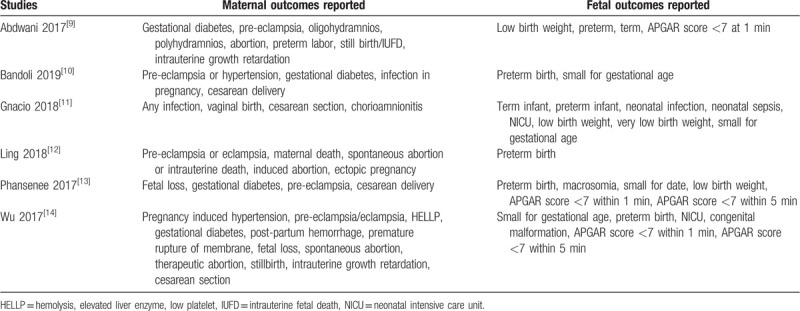
Outcomes reported.

### 2.6 Outcomes to be analyzed in this study

The following maternal complications were analyzed in this study:

(1) Pre-eclampsia/eclampsia;
(2) Stillbirth;
(3) Fetal loss;
(4) Abortion including spontaneous and therapeutic abortions;
(5) Cesarean section;
(6) Intrauterine growth retardation;
(7) Gestational diabetes.

The following fetal complications were analyzed in this study:

(1) Preterm birth;
(2) Infants who were small for gestational age;
(3) Infants with low birth weight;
(4) Admission to the neonatal intensive care unit (NICU);
(5) Newborns with an APGAR score of <7 within 1 and 5 minutes, respectively.

### 2.7 Statistical analysis

The RevMan software 5.3 (latest version) was the most suitable analytical software for this analysis. Data were represented by risk ratio (RR) with 95% confidence interval (CI).

Meta-analyses are prone to heterogeneity. In this analysis, heterogeneity was assessed by the *Q* statistic test. A subgroup analysis with a *P*-value less or equal to .05 was considered as statistically significant. Heterogeneity was also assessed by the *I*^2^ statistic test, whereby, the heterogeneity was increased with an increasing value of *I*^2^.

A fixed (*I*^2^ < 50%) or a random (*I*^2^ > 50%) effects statistical model was used based on the value of heterogeneity (*I*^2^).

Sensitivity analysis and publication bias were also assessed.

### 2.8 Compliance with ethical guideline

This analysis only involved data which were extracted from previously published studies. This study does not involve any experiment with humans or animals carried out by any of the authors. Therefore, an ethical approval was not required.

## 3 Results

### 3.1 Searched outcomes

This search process was carried out based on the PRISMA guideline.^[8]^ A total number of 4026 publications were obtained. Based on an initial assessment of the abstracts and titles, 3854 articles were directly eliminated due to irrelevance. One hundred seventy-two (172) full-text articles were assessed for eligibility.

Further studies were eliminated due to the following reasons:

(1) They were literature reviews, meta-analyses or systematic reviews (n = 8);
(2) They were case studies (n = 23);
(3) A control group was absent (n = 37);
(4) A comparison was missing between SLE and non-SLE pregnant women (n = 26);
(5) They were studies that were published during or before the year 2016 (n = 30);
(6) They did not report the relevant outcomes (n = 9);
(7) They were published in a different language apart from English (n = 3);
(8) They were duplicated studies (30).

**Figure 1:**
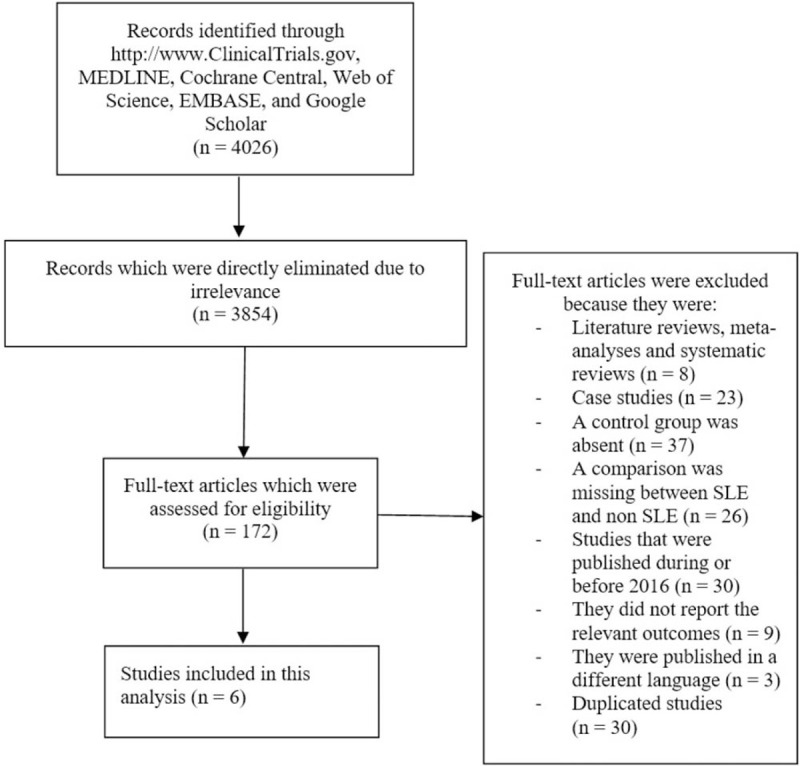
Flow diagram representing the study selection.

Finally, only 6 studies^[9–14]^ were selected for this analysis as shown in Figure 1.

### 3.2 Main and baseline features of the studies

All the studies were observational cohorts. One study enrolled participants from the year 1987 to 2013. The other studies had an enrollment time period between years 2001 and 2017 as shown in Table 2.

**Table 2:**
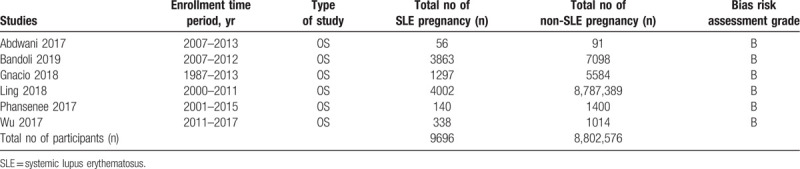
General features of the studies.

A total number of 8,812,272 participants were included in this analysis, consisting of 9696 SLE-associated pregnancy.

Based on an assessment of the methodological quality of the studies, a grade B was allotted to all the studies implying a moderate risk of bias.

Table 3 lists the baseline features of the pregnant women with and without SLE. Mean age varied from 19.0 to 31.0 years. Pregnant women who smoked (7.30%–12.7%), maternal body mass index and the number of prenatal care visits were also listed.

**Table 3:**
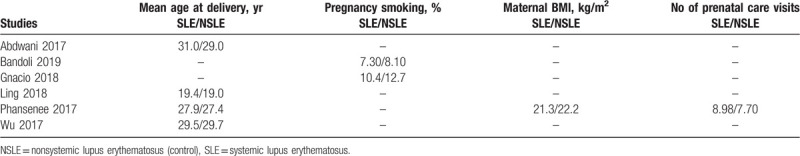
Baseline features of the pregnant women.

### 3.3 Maternal complications associated with SLE

**Figure 2:**
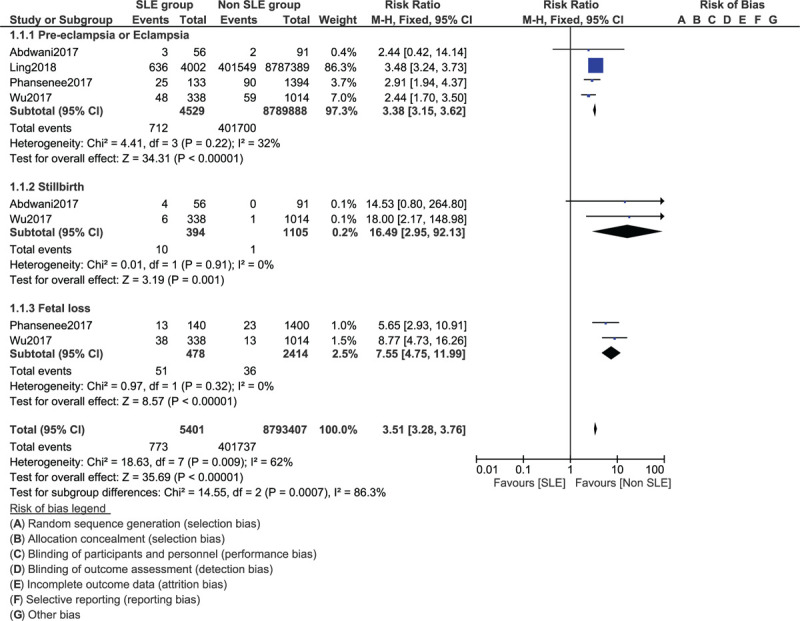
Maternal complications related to systemic lupus erythematosus (Part I).

Based on an analysis of recently published studies (2017–2019), pre-eclampsia/eclampsia was significantly higher in pregnant women with SLE (RR: 3.38, 95% CI: 3.15–3.62; *P* = .00001) as shown in Figure 2. SLE was also associated with an increased risk of stillbirth (RR: 16.49, 95% CI: 2.95–92.13; *P* = .001) and fetal loss (RR: 7.55, 95% CI: 4.75–11.99; *P* = .00001) as shown in Figure 2.

**Figure 3:**
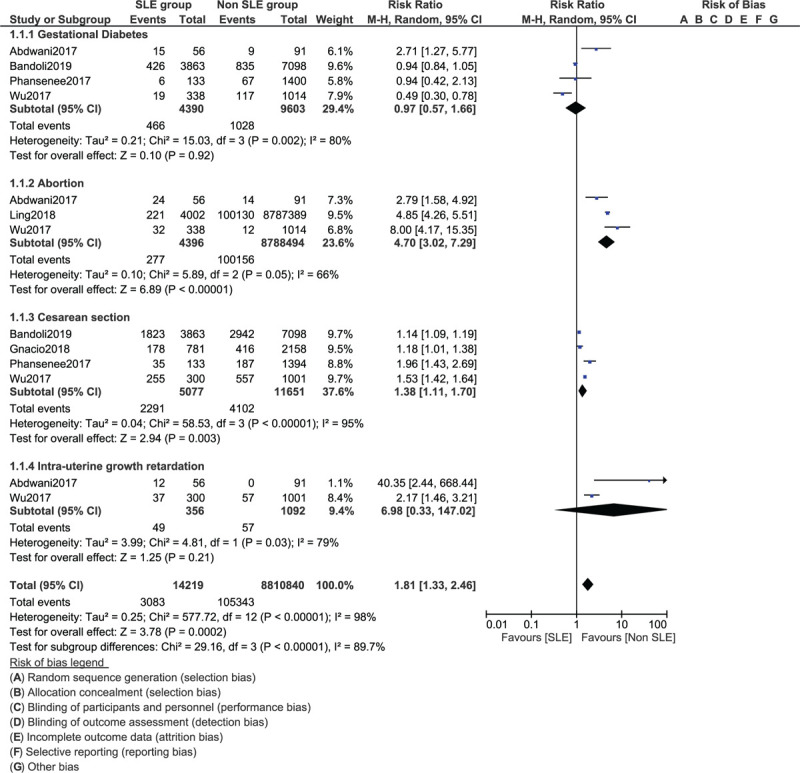
Maternal complications related to systemic lupus erythematosus (Part II).

Abortion (RR: 4.70, 95% CI: 3.02–7.29; *P* = .00001) and the risk for cesarean section due to complications (RR: 1.38, 95% CI: 1.11–1.70; *P* = .003) were also significantly higher in pregnant women with SLE as shown in Figure 3. However, intrauterine growth retardation (RR: 6.98, 95% CI: 0.33–147.02; *P* = .21) and gestational diabetes (RR: 0.97, 95% CI: 0.57–1.66; *P* = .92) were similar in both groups (Fig. 3).

### 3.4 Fetal complications associated with SLE

**Figure 4:**
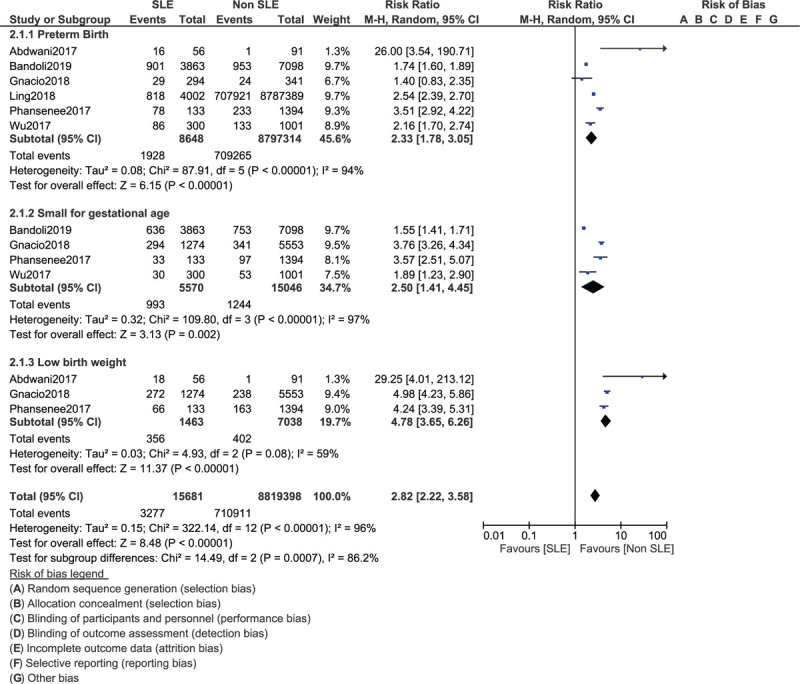
Fetal complications related to mothers with systemic lupus erythematosus (Part I).

Based on recently published studies (2017–2019), fetal complications including preterm birth (RR: 2.33, 95% CI: 1.78–3.05; *P* = .00001), infants who were small for gestational age (RR: 2.50, 95% CI: 1.41–4.45; *P* = .002) and infants with low birth weight (RR: 4.78, 95% CI: 3.65–6.26; *P* = .00001) were also significantly higher in newborns from mothers with SLE as shown in Figure 4.

**Figure 5:**
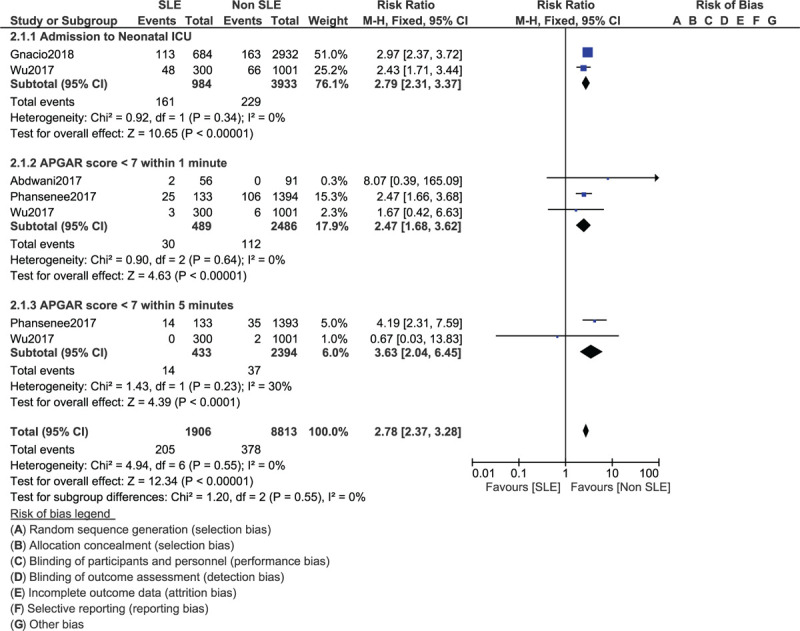
Fetal complications related to mothers with systemic lupus erythematosus (Part II).

The risk of newborns who were admitted to the NICU (RR: 2.79, 95% CI: 2.31–3.37; *P* = .00001), newborns with an APGAR score <7 within 1 minute (RR: 2.47, 95% CI: 1.68–3.62; *P* = .00001) and newborns with an APGAR score <7 within 5 minutes (RR: 3.63, 95% CI: 2.04–6.45; *P* = .0001) were significantly highly associated with SLE as shown in Figure 5.

**Figure 6:**
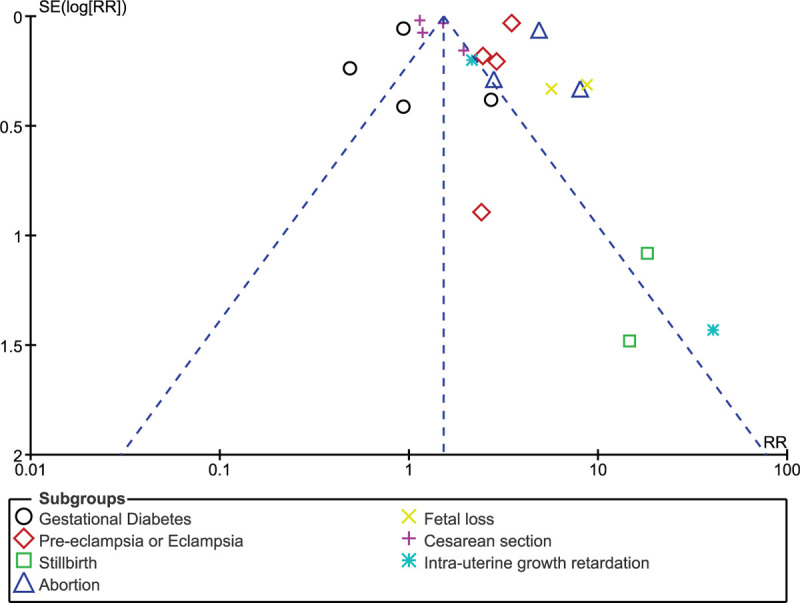
Funnel plot representing publication bias (maternal complications).

**Figure 7:**
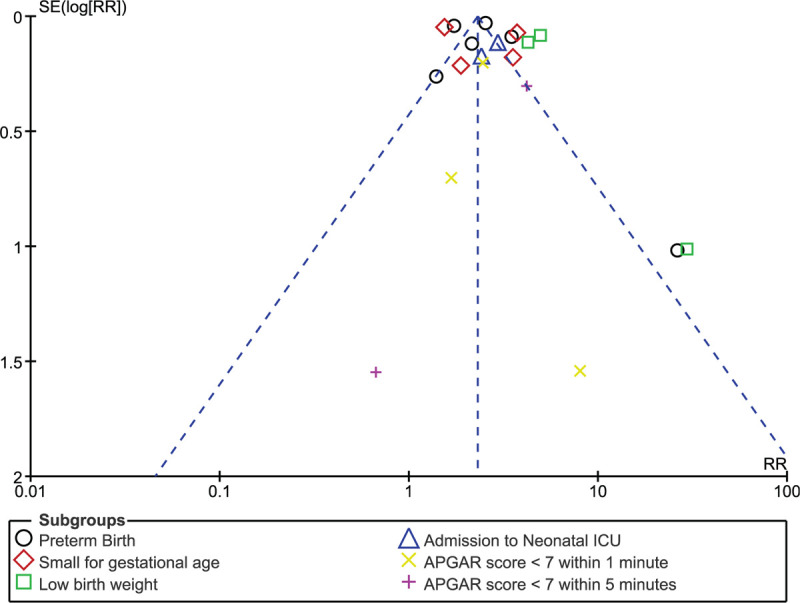
Funnel plot representing publication bias (fetal complications).

Consistent results were obtained when each study was by turn excluded followed by a new analysis each time. Low evidence of publication bias was observed in certain subgroups among the studies which assessed maternal and fetal outcomes associated with pregnant women with and without SLE and this was represented in Figures 6 and 7.

A summarized table representing the results has been provided as Table 4.

**Table 4:**
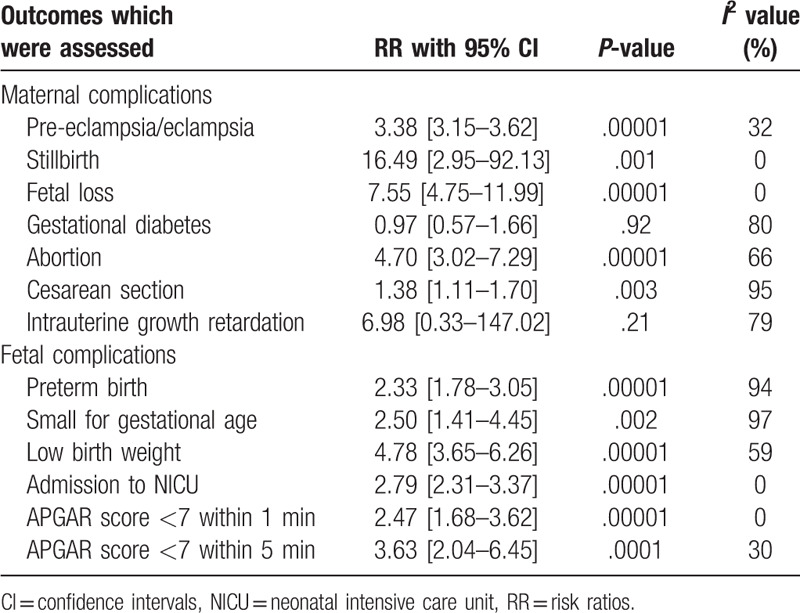
Summarized results.

## 4 Discussion

A meta-analysis based on the impact of SLE on maternal and fetal outcomes was previously published by Bundhun et al.^[5]^ The authors clearly showed SLE-associated pregnancies to be considered as high risk pregnancies. However, only studies published during and before the year 2016 were included in their analysis.

Recently, modifications have been carried out in guidelines for the better treatment and management of pregnant women with SLE or other autoimmune disorders.^[6]^ During these recent years, several new studies based on pregnancy in SLE patients were published. Hence, considering the most recent publications (2017–2019),^[9–14]^ we have systematically carried out this analysis.

Our current analysis showed that maternal complications such as pre-eclampsia/eclampsia, fetal loss, stillbirth, and abortion were significantly higher in pregnant women with SLE. The risk for cesarean section was also increased in such patients.

In addition, fetal complications such as preterm birth, infants who were small for gestational age, infants who had a low birth weight, newborns admitted to the NICU and newborns with an APGAR score <7 within 1 and 5 minutes, respectively were significantly higher in infants born from mothers with SLE.

Our current analysis has complete support from other recently published studies. A retrospective study conducted in Southern China also showed with full evidence, the association of adverse pregnancy outcomes including pregnancy loss and preterm delivery in women with SLE.^[15]^ The authors also stated that umbilical artery Doppler was a good method to monitor these adverse pregnancy complications during the third trimester of pregnancy.

Apart from these pregnancy outcomes, other studies have shown maternal SLE to be associated with dyslexia, attention deficit, and speech disorders in offspring due to developmental issues.^[16]^

Because reproductive issues are common in women with SLE, pre-pregnancy assessment to identify highly at risk women, and counseling advice should be given to women if pregnancy is to be avoided.^[17]^

Even if updated guidelines were published for the management of pregnant SLE patients, another recent study showed that even with low molecular weight heparin and aspirin use during pregnancy, maternal and perinatal complications occurred frequently^[18]^ which might not be a positive response to therapy. However, other measures such as a predictive model for fetal loss to identify high risk pregnancies, as shown in a Chinese retrospective study might be helpful to these women with SLE.^[19]^

## 5 Limitations

This analysis had the following limitations: several baseline features of the participants were not listed since they were not reported in the original studies. Therefore, important information such as the duration of disease were not available. There was insufficient information about the follow-up of these patients and the medications used during this pregnancy period, and this might have had an impact on the outcomes. In addition, since this analysis involved observational data, confounding variables and bias were observed. Because this analysis was based on studies which were published after the year 2016, the total number of studies that satisfied the inclusion and exclusion criteria was less. However, we could not include studies that were published during or before the year 2016 since another previous meta-analysis was already based on studies published before the year 2016.

## 6 Conclusions

Based on the most recent studies (2017–2019), we could conclude that maternal and fetal complications were significantly higher in SLE-associated pregnancy. Therefore, SLE should still be considered a severe risk factor for pregnancy.

## Author contributions

**Conceptualization:** Wen Rong He, Hua Wei.

**Data curation:** Wen Rong He, Hua Wei.

**Formal analysis:** Wen Rong He, Hua Wei.

**Funding acquisition:** Wen Rong He, Hua Wei.

**Investigation:** Wen Rong He, Hua Wei.

**Methodology:** Wen Rong He, Hua Wei.

**Project administration:** Wen Rong He, Hua Wei.

**Resources:** Wen Rong He, Hua Wei.

**Software:** Wen Rong He, Hua Wei.

**Supervision:** Wen Rong He, Hua Wei.

**Validation:** Wen Rong He, Hua Wei.

**Visualization:** Wen Rong He, Hua Wei.

**Writing – original draft:** Wen Rong He, Hua Wei.

**Writing – review & editing:** Wen Rong He, Hua Wei.
